# Closed‐Loop AI Systems for Arrhythmia Management: Toward Predictive, Personalized, and Safe Cardiac Care

**DOI:** 10.1002/clc.70468

**Published:** 2026-09-08

**Authors:** Mohammed AbuBaha, Yousef I. Barqawi, Bara AbuBaha, Salsabeel Bishawi, Duma Hamada, Mohammed Saleh, Sarah Saife, Yahia AlJallad, Elias Salah, Anas Salah, Hossam Salameh

**Affiliations:** ^1^ Department of Medicine An‐Najah National University Nablus Palestine; ^2^ Department of Medicine Al‐Quds University Jerusalem Palestine

**Keywords:** adaptive control systems, arrhythmias, artificial intelligence, cardiac arrhythmias, clinical decision support, closed‐loop control, continuous ECG monitoring, electrophysiology, implantable cardiac devices, reinforcement learning

## Abstract

**Background:**

Cardiac arrhythmias impose a substantial global health and economic burden. Despite advances in electrophysiology and device‐based therapy, management still depends on episodic monitoring and delayed decision‐making, leaving a persistent gap between detection and intervention that is widest for intermittent and asymptomatic rhythm disturbances.

**Objective:**

This narrative review traces the shift from open‐loop arrhythmia care to closed‐loop, AI‐assisted systems that couple continuous sensing, automated decision‐making, and therapeutic actuation within a feedback‐controlled architecture. Its contribution is to reframe arrhythmia AI around the control loop and its safety constraints rather than around detection accuracy alone, addressing a gap left by reviews that focus on classification algorithms or digital‐twin modeling.

**Methods:**

We synthesized evidence from landmark device‐programming trials, foundational work in control theory and machine learning, and current regulatory guidance to define the principles, technological basis, and system‐level requirements of closed‐loop arrhythmia care. As a narrative review, it emphasizes control logic, safety constraints, feedback, and clinical integration rather than algorithmic performance in isolation.

**Results:**

Device‐programming trials show that conservative timing, hierarchical escalation, and defined safety boundaries reduce inappropriate therapy and can improve survival, establishing the control principles on which AI systems must build. Machine learning extends these principles to scalable monitoring and adaptive decision‐making but introduces concerns of stability, transparency, bias, and performance drift. Safe implementation therefore requires dependable sensing, bounded adaptation, supervisory control, and lifecycle evaluation.

**Conclusions:**

Closed‐loop AI extends established electrophysiologic control principles rather than replacing clinical judgment. Embedded within transparent, governed architectures with bounded adaptation and human oversight, such systems may improve the personalization, safety, and outcomes of arrhythmia care.

## Graphical abstract

This review reframes arrhythmia AI around the control loop and its safety constraints, not accuracy alone. Randomized device‐programming trials supply the principles (conservative timing, hierarchical escalation, bounded actuation) that closed‐loop AI must inherit. Within governed architectures with bounded adaptation and human oversight, such systems may augment, not replace, clinical judgment.

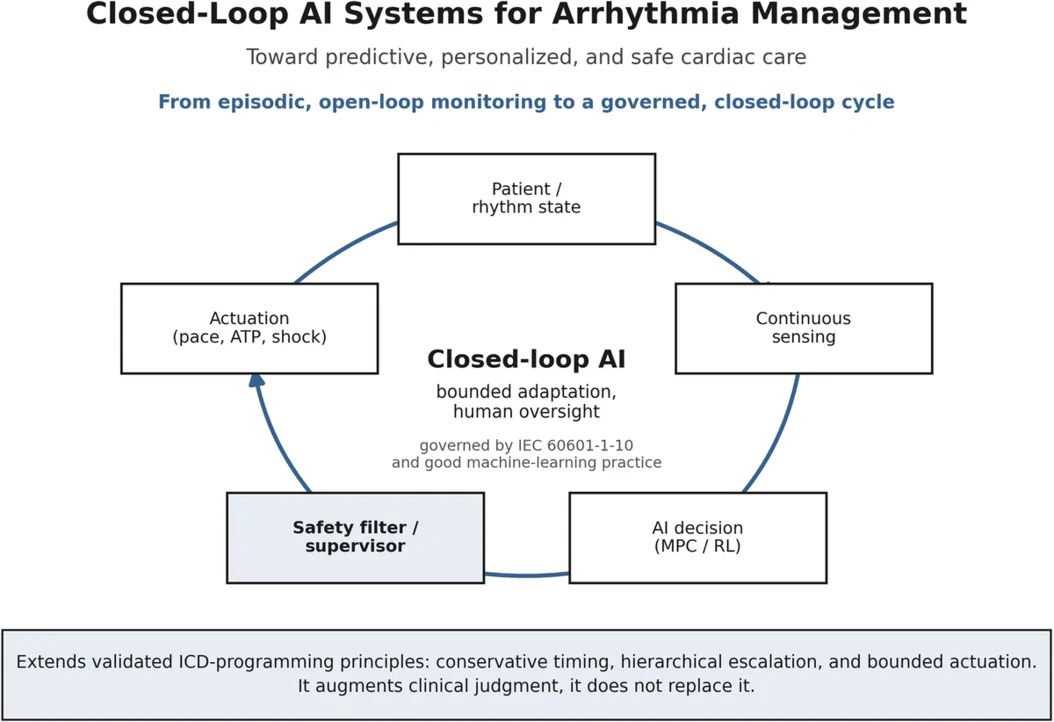

**Abbreviations**
ADVANCE III: Avoid Delivering Therapies for Nonsustained Arrhythmias in ICD Patients III
AF: Atrial Fibrillation
AI: Artificial Intelligence
ATP: AntiTachycardia Pacing
BIOSync CLS: BIOSync Closed‐Loop Stimulation
CNN: Convolutional Neural Network
DL: Deep Learning
ECG: Electrocardiogram/Electrocardiography
FDA: U.S. Food and Drug Administration
ICD: Implantable Cardioverter‐Defibrillator
LSTM: Long Short‐Term Memory
MADIT‐RIT: Multicenter Automatic Defibrillator Implantation Trial–Reduce Inappropriate Therapy
MPC: Model Predictive Control
PainFREE Rx II: Pacing Fast Ventricular Tachycardia Reduces Shock Therapies II
RL: Reinforcement Learning
RNN: Recurrent Neural Network
U.S.: United States

## 1 Introduction

Cardiac arrhythmias are among the most consequential cardiovascular disorders worldwide, contributing substantially to stroke, heart failure, sudden cardiac death, and recurrent hospitalization. Advances in electrophysiology and device‐based therapy have improved outcomes, yet routine care still relies on intermittent surveillance, short‐term ECG, Holter monitoring, and scheduled device interrogation. These methods capture rhythm only in brief, pre‐specified windows and frequently miss arrhythmias that are transient, context‐dependent, or early in their course, delaying diagnosis and treatment. This detection–intervention gap motivates continuous monitoring and automated decision‐making that operate independently of periodic clinical encounters.

Artificial intelligence has transformed automated ECG interpretation. Convolutional and recurrent neural networks, including long short‐term memory models, now classify arrhythmias with accuracy that exceeds conventional rule‐based algorithms [1]. In a landmark study, a deep neural network achieved cardiologist‐level performance in classifying rhythms from single‐lead ambulatory ECG [2]. These advances allow detection algorithms to run unobtrusively within wearable and ambulatory devices, enabling continuous rhythm monitoring outside the clinic.

Detection alone, however, does not close the therapeutic loop. In a closed‐loop architecture, sensed signals drive algorithmic decisions that trigger an intervention whose physiological effect is then fed back to inform subsequent action. Early experimental systems already couple AI‐based detection to actuation: an untethered, AI‐supported monitor has demonstrated real‐time arrhythmia detection and classification suitable for such loops [3]. Implantable pacemakers and cardioverter‐defibrillators (ICDs) are the established clinical embodiment, autonomously sensing intracardiac signals and delivering therapy by preset rules. Randomized programming trials show that disciplined automation (delayed detection and antitachycardia‐pacing‐first escalation) reduces unnecessary interventions and can improve survival [4, 5].

Adaptive, learning‐based control extends what closed‐loop systems can do. Reinforcement learning (RL) revises control policies from ongoing physiological feedback, offering a route to personalize and retime therapy; computational studies show that RL controllers can learn to modulate cardiovascular function as conditions change [6]. Deploying adaptive algorithms in a safety‐critical setting, however, raises pointed concerns about stability, generalizability, and unintended behavior, concerns that frame much of this review.

As these systems gain autonomy, interpretability and regulatory compliance become preconditions for clinical deployment, not afterthoughts.

Explainability methods applied to ECG deep‐learning models highlight the waveform features driving a prediction, strengthening clinician trust [7]. Professional societies and regulators emphasize that cardiovascular AI must remain transparent, accountable, and under human control wherever its outputs inform treatment [8]. Closed‐loop arrhythmia management is therefore only partly a computational problem; it is equally a systems‐engineering and governance challenge.

A growing literature reviews arrhythmia AI, but most accounts center on detection algorithms or digital‐twin modeling and stop short of the control logic, actuation safety, and lifecycle governance that separate algorithmic sensing from accountable therapy. This review addresses that gap by organizing the field around the control loop itself. To structure the discussion we use a Closed‐Loop AI Control Stack of five interacting layers: continuous physiological sensing, algorithmic decision‐making, therapeutic actuation, feedback‐driven adaptation, and supervisory governance with human oversight. The stack is intended as an organizing and teaching framework rather than a new formalism; it operationalizes, for arrhythmia care, the principles already codified in the international standard for physiologic closed‐loop controllers (IEC 60601‐1‐10) and in regulatory expectations for adaptive software, including good machine‐learning practice and predetermined change‐control plans. Throughout, we treat safety, accountability, and clinical integration as the defining requirements of closed‐loop AI in arrhythmia management.

## 2 From Open‐Loop Automation to Closed‐Loop Arrhythmia Care

Arrhythmia care has traditionally followed an open‐loop workflow in which sensing, interpretation, and treatment occur as separate, time‐displaced steps: clinicians review short ECGs, Holter recordings, or scheduled device interrogations and adjust therapy retrospectively. This approach has advanced diagnosis and treatment but systematically misses arrhythmias that are transient, context‐dependent, or asymptomatic. The cost is clearest in atrial fibrillation (AF), where unrecognized episodes delay anticoagulation and permit preventable thromboembolism. In the STROKESTOP screening study of 7173 individuals aged 75–76 years, intermittent ECG over 2 weeks identified previously unknown AF in 3.0%, roughly a fourfold increase over the 0.5% detected on a single recording, exposing the inadequacy of episodic surveillance for intermittent arrhythmia [9].

Wearable and consumer technologies have pushed rhythm surveillance beyond the clinic. The Apple Heart Study enrolled 419,297 participants; 0.52% received an irregular‐pulse notification, and among those who returned an analyzable ECG patch, AF was confirmed in 34%, with a positive predictive value of 0.84 for the notification [10]. The low notification rate and the modest confirmation yield illustrate both the promise and the limits of consumer screening: such tools generate large volumes of presumptive findings that still require clinical adjudication. Deep‐learning interpretation converts this ambulatory data into actionable rhythm classification, and the demonstration that neural networks can match cardiologists on single‐lead ECG underpins continuous AI‐enabled monitoring pipelines [2].

Detection alone does not close the loop. A closed‐loop system adds decision logic and actuation bound by feedback constraints, and the ICD is its clearest clinical embodiment: it continuously monitors intracardiac signals and discharges only when automatic detection criteria are met. The ICD era also delivered an enduring lesson, automation harms patients when its control logic is insufficiently conservative. In MADIT‐RIT (1500 patients), high‐rate and delayed‐therapy programming reduced first inappropriate therapy from 20% to roughly 4% (hazard ratios 0.21 and 0.24 vs. conventional programming) and lowered all‐cause mortality (hazard ratio 0.45 for high‐rate programming) [4]. The benefit came not from faster response but from governing when and how therapy escalates.

Subsequent programming trials confirmed the principle. In ADVANCE III (1902 patients), a long detection window (30 of 40 intervals) versus standard detection (18 of 24) reduced inappropriate shocks (5.1 vs. 11.6 per 100 person‐years; incidence rate ratio 0.55) without increasing syncope or mortality, showing how a temporal constraint functions as a safety guardrail [5]. In PainFREE Rx II (634 patients), empirical antitachycardia pacing (ATP) terminated roughly 70% of fast ventricular tachycardia episodes (188–250 bpm) without shocks and reduced shock burden, with no excess syncopem, evidence for a hierarchical, least‐harm‐first escalation ladder [11]. Together these trials establish that closed‐loop arrhythmia care is most effective when thresholds, delays, and escalation are tuned for protection rather than speed.

Pacing has evolved along the same line, toward physiologically responsive feedback rather than static rate adaptation. In BIOSync CLS (127 patients with recurrent reflex syncope and tilt‐induced asystole), dual‐chamber pacing with closed‐loop stimulation reduced the 2‐year syncope recurrence from 68% to 22% (a 77% relative reduction) and the trial was stopped early for efficacy [12]. The residual 22% recurrence is itself instructive: feedback‐informed actuation delivers clinical benefit within tightly specified policies but does not eliminate failure, reinforcing the need for conservative defaults, predictable behavior, and validated intervention pathways in any AI‐enabled successor.

Despite real breakthroughs in detection and prediction, most deployed systems remain “open‐loop AI”: they emit alerts or risk scores with no built‐in mechanism for safe actuation or supervisory control. Monitoring improves, but the gap between sensing and therapy persists, and clinicians must translate algorithmic output into action under time pressure. As Armoundas et al. emphasize, trustworthy clinical AI depends on transparency, clinical evaluation, and governance that guarantee safety, auditability, and human oversight wherever outputs drive treatment [8]. Closing the loop is therefore not a matter of stronger models alone; it requires clinically grounded control architectures that define when an algorithm may act, when it must defer, and how its performance is monitored over time.

## 3 The Closed‐Loop AI Paradigm

A closed‐loop AI system treats sensing, decision‐making, and therapy as one continuously interacting cycle rather than discrete steps. In its canonical form it is a sensor–processor–actuator configuration updated by feedback, so that each intervention is informed by the physiological state it produces [13]. Arrhythmia care makes this architecture especially demanding: rhythm disturbances can escalate within seconds, vary by context, and require immediate action to prevent downstream harm.

The cycle begins with sensing, continuous ECG or intracardiac electrograms drawn increasingly from ambulatory wearables and implantable devices. AI classifiers convert these time series into rhythm labels, episode probabilities, or risk estimates, and neural networks already match cardiologist‐level rhythm classification at scale [2]. What distinguishes a closed‐loop system from a classifier is the structured pathway linking interpretation to a controlled response and treating the evolving rhythm state as feedback.

Arrhythmia control is a time‐critical problem in a nonlinear biological system, where excessive intervention harms and delayed intervention can be fatal. Control theory addresses this trade‐off through stability, constraint satisfaction, and predictable behavior under uncertainty. Model predictive control (MPC) is particularly well matched to therapeutic automation: at each step it solves a finite‐horizon optimization over a model of the patient–device dynamics, minimizing a cost—such as deviation of rate or rhythm from a target, subject to hard constraints, then applies only the first action before re‐optimizing as new measurements arrive. The receding‐horizon structure encodes safety directly, because pacing‐rate limits, energy ceilings, and charge‐time bounds enter the controller as explicit constraints rather than post‐hoc checks [14]. This makes MPC a natural substrate for cardiovascular interventions that must remain within validated physiological and device envelopes.

Electrophysiology provides the strongest clinical evidence that constrained closed‐loop control works. The device‐programming trials discussed above (MADIT‐RIT and ADVANCE III) show that conservative timing and escalation reduce inappropriate therapy and, in MADIT‐RIT, improve survival, whereas aggressive detection worsens outcomes [4, 5]. For AI‐enabled loops the lesson is direct: performance is not reaction speed but fidelity to timing constraints and escalation logic that prevent harm.

Beyond fixed control logic, closed‐loop systems increasingly incorporate learning that adapts to individual physiology. Reinforcement learning (RL) formalizes therapy as a control policy mapping an observed state (rhythm features and hemodynamic surrogates) to an action such as a pacing‐rate change, an ATP burst, or deferral, chosen to maximize a cumulative reward that credits rhythm stabilization and penalizes inappropriate or high‐energy therapy. In computational studies, RL controllers learned stimulation strategies that stabilized cardiovascular dynamics under changing conditions [6]. The appeal for arrhythmia care is that substrates, triggers, and treatment responses vary widely between patients and within the same patient over time, which static rules capture poorly.

Learning from feedback also raises clinical risk when adaptation is not bounded by explicit safety constraints. RL is vulnerable to harmful exploration, poor generalization under distribution shift, and instability outside its training distribution; in medicine, where live trial‐and‐error is unacceptable, policies must instead be assessed by off‐policy evaluation on logged data within a risk‐aware framework [15]. The practical resolution is a bounded‐RL safety envelope: a learned policy proposes actions, but a model‐based or rule‐based supervisor (an MPC layer or validated programming limits) vetoes any action outside approved physiological and device bounds before it reaches the patient. Because a wrong adaptation in arrhythmia care can mean an inappropriate shock or withheld therapy, this bounded design is far more clinically feasible than unrestricted autonomy.

Clinical feasibility depends less on classification accuracy than on whether a system operates safely, transparently, and under accountable oversight. The supervisory layer is where this is enforced: it decides when an algorithm may act, when it must defer to clinician review, and how escalation is constrained by validated rules. As applications in electrophysiology move from decision support toward autonomy, AI output must be interpreted in clinical context and embedded in workflows (device clinics, remote‐monitoring pathways, and the electrophysiology laboratory) that preserve safety and accountability [16].

Interpretability is equally central, because clinicians adopt automated decisions only when they can scrutinize them. Explainable‐AI methods for ECG deep learning expose the features that drive a classification, and the field's progress depends on meeting clinician needs for transparency and trust rather than pursuing accuracy alone [7]. Auditability, clinical scrutiny, and human oversight remain mandatory wherever algorithmic output affects treatment, again situating closed‐loop arrhythmia management as a governance and systems‐engineering problem, not merely a computational one.

## 4 Technological Foundations of Closed‐Loop AI Systems for Arrhythmia Management

Closed‐loop AI for arrhythmia rests on an integrated technological stack: sensing hardware, signal processing, computation, and therapeutic actuation. Its foundation is continuous acquisition of cardiac electrical activity, without which timely detection and intervention are impossible. Wearable sensors, patch‐based ECG systems, and implantable loop recorders now deliver long‐term monitoring with progressively higher signal fidelity outside the clinic.

Continuous ECG monitoring markedly increases detection of intermittent and asymptomatic arrhythmias relative to episodic strategies, which is why it anchors closed‐loop architectures [9, 17]. Ambulatory and implantable signals remain vulnerable to noise, motion artifact, baseline drift, and lead variability, so preprocessing and signal conditioning are prerequisites for reliable analysis. Early pipelines relied on handcrafted features (RR intervals, P‐wave morphology, frequency‐domain metrics) whereas deep models now learn discriminative features directly from raw waveforms. End‐to‐end convolutional networks achieve high‐quality arrhythmia classification with minimal preprocessing [18], and comparable performance across datasets and device configurations supports their use in real‐world monitoring [2].

Embedding models in wearable and implantable hardware imposes hard limits on latency, energy, and reliability, forcing compact architectures and efficient inference. Model compression, quantization, and on‐device (edge) computing enable real‐time arrhythmia detection without continuous cloud connectivity [19]. Hybrid designs are increasingly common: local processing handles initial detection, while more complex or longitudinal analyses are offloaded to external or cloud platforms when bandwidth and latency permit, an architecture exemplified by AI‐enabled ECG analysis that identifies patients with paroxysmal AF from sinus‐rhythm recordings [20].

Actuation is what separates a closed‐loop system from a purely diagnostic one. Invasive systems act through pacing, ATP, cardioversion, or defibrillation; non‐invasive systems are limited to alerts, medication reminders, or clinician notification. The safety and efficacy of actuation rest on electrophysiologic principles established over decades of device experience, and the landmark programming trials show that outcomes depend on therapy timing, escalation logic, and energy strategy, not on detection accuracy alone [4, 5]. Algorithmic decisions must therefore pass through validated therapeutic rules before reaching the patient.

Feedback is what makes a system closed‐loop: it evaluates the effect of a therapy and conditions the next action on that result. Signals may be direct (rhythm termination, rate normalization, recurrence) or indirect surrogates such as heart‐rate variability or activity [6]. Because physiological feedback is often delayed, noisy, and nonstationary, robust controllers combine short‐term response with longer‐term outcome tracking to steer behavior without overreacting to transient fluctuations.

Finally, the technological stack must support continuous validation and post‐deployment monitoring. Deployed models degrade through data drift, population change, and device variation, and regulators and professional societies increasingly require mechanisms for auditability, controlled model updating, and ongoing evaluation of safety and effectiveness [8, 21]. Design choices must therefore anticipate not only first deployment but years of clinical operation.

## 5 Governance, Ethics, and Regulatory Considerations in Closed‐Loop AI Arrhythmia Systems

As closed‐loop systems move from detection toward semi‐autonomous or autonomous intervention, governance and accountability become as decisive as model accuracy [22]. Unlike fixed‐logic devices, AI‐enabled systems can update decision thresholds over time and emit probabilistic outputs that shape therapy. In arrhythmia care, where automated pacing, ATP, or defibrillation carries immediate and serious consequences, governance (not raw accuracy) is often the operative determinant of safety.

The central ethical question in closed‐loop care is the gradual transfer of therapeutic authority from clinicians to algorithms. Automation can shorten response times and reduce workload, yet it blurs responsibility when an algorithm‐driven action causes harm. Armoundas et al. argue that any AI affecting patient care must be transparent, clinically validated, and operated under accountable oversight wherever its outputs inform treatment [8]. This translates into concrete engineering requirements: clinician override, comprehensive state logging, and interpretable records of when and why each action was triggered.

Regulation is adapting to software as a medical device (SaMD) and to adaptive learning systems. A static, one‐time approval is ill‐suited to algorithms that change after deployment, and guidance has shifted toward lifecycle oversight, predetermined change‐control plans, and post‐market evaluation [23]. Yet analyses of cleared AI tools show that most are narrow and task‐specific, approved through pathways that do not yet capture adaptive closed‐loop behavior, evidence that governance standards must mature as autonomy increases [22].

Transparency and explainability are further prerequisites. Clinicians will not adopt AI‐driven therapeutic loops they cannot interrogate, particularly in high‐stakes electrophysiology that demands rapid decisions while guarding against unnecessary therapy. Deep‐learning ECG systems must therefore satisfy interpretability and reliability alongside performance, because trust and post‐hoc review are essential to safe use [7]. In a closed loop, explanation matters even more: it supports accountability, enables error detection, and helps clinicians recognize when automation has become inappropriate or harmful.

Bias and generalizability are safety‐critical governance problems. When training data underrepresent certain populations or encode structural inequities, an algorithm can perform unevenly across demographic groups, producing unequal detection and unequal therapy. Obermeyer et al. showed that a seemingly reasonable proxy in a widely used health algorithm generated large racial disparities, demonstrating that bias can persist even when a model appears accurate by conventional metrics [24]. In closed‐loop arrhythmia care, fairness assessment is thus a clinical safety requirement rather than an optional add‐on, because biased inference yields biased therapy.

Human–AI interaction adds a further layer of risk through two distinct failure modes. Automation bias is over‐reliance: clinicians accept algorithmic output as correct and omit independent verification, an effect that intensifies with cognitive load and with the difficulty of checking the recommendation [25]. Alert fatigue is the opposite desensitization: excessive or poorly designed alerts erode attention until even valid warnings are ignored. The two pull system design in different directions (one demands easier verification and preserved clinician engagement, the other demands fewer, better‐targeted alerts) and a safe closed‐loop interface must address both simultaneously.

Post‐deployment monitoring is essential because closed‐loop systems operate in environments that change over time. Sensing hardware degrades, patient populations shift, and clinical practice evolves, each eroding model performance. Finlayson et al. identify dataset shift as a principal vulnerability of clinical AI and call for explicit detection and mitigation [26]. In a closed loop, drift monitoring must extend beyond classification accuracy to therapy‐triggering behavior, escalation rates, and downstream outcomes, because harm can emerge even while diagnostic performance appears stable.

Governance must also encompass cybersecurity and data protection. Closed‐loop systems depend on wireless links, telemedicine, and cloud exchange, widening the attack surface and exposing sensitive data to interception. Das et al. review vulnerabilities in cardiac implantable electronic devices and argue that cybersecurity is a patient‐safety issue, not merely a technical one [27]. Where networked pathways connect sensing to actuation, security controls become an inseparable part of clinical governance.

Together, governance, ethics, and regulation form the foundation for closed‐loop AI in arrhythmia management. Advances in sensing and inference will not improve care unless they are embedded in accountable architectures that provide transparency, fairness auditing, cybersecurity, and lifecycle oversight. With these safeguards, closed‐loop autonomy can become a structured path to safer, more personalized arrhythmia care; without them, it risks introducing the very harms it aims to prevent.

## 6 Future Directions in Closed‐Loop AI for Arrhythmia Management

The future role of closed‐loop AI in arrhythmia management will depend less on algorithmic refinement than on resolving the constraints around it. Continuous monitoring and automated interpretation have advanced detection, but converting these capabilities into reliable closed‐loop systems remains unsolved. The central task is to couple AI‐based decision‐making to therapeutic action safely and durably under real‐world conditions.

A central goal is personalization that remains reliable and safe. Arrhythmia burden, triggers, and treatment response vary between patients and shift over time with disease progression and therapy. Reinforcement‐learning approaches could tune detection thresholds and intervention timing to an individual's physiology, as computational controllers that adapt cardiovascular regulation from feedback suggest [6]. As emphasized above, such adaptation is clinically acceptable only within a bounded safety envelope; uncontrolled learning risks instability and unsafe behavior.

Prospective clinical evaluation is the next priority. Most AI arrhythmia tools have been validated only retrospectively; few have been tested in randomized trials with patient‐centered outcomes. Because closed‐loop systems run continuously and adapt over time, conventional trial designs may not capture their effects, and AI‐specific reporting and evaluation standards are needed [28]. Trials of closed‐loop arrhythmia systems should report not only detection accuracy but therapy appropriateness, safety events, and long‐term outcomes such as syncope, inappropriate shocks, and survival.

Human interaction will shape clinical success. Even highly automated systems coexist with clinicians who retain ultimate responsibility for oversight, so explanations must align with human reasoning rather than report abstract metrics [29]. Future arrhythmia systems should communicate their confidence and the rationale for each suggested or delivered intervention, especially when automation affects therapy.

Equity and generalizability remain pressing for deployment. Models trained on small or homogeneous datasets may underperform across diverse populations, creating disparities in detection and intervention [30]. In a closed loop, unequal performance becomes unequal exposure to therapy, reinforcing the need for representative training data and continuous, privacy‐preserving performance auditing.

Finally, durable deployment depends on sustained monitoring, governance, and‐critically‐ integration into real clinical workflows. Performance deteriorates as populations, hardware, and practice change, with distribution shift a leading cause of failure in deployed clinical AI [26]. Practical adoption will hinge on fitting these systems into existing infrastructure: device clinics and remote‐monitoring services, alarm‐management and triage pathways, electronic health record interoperability, and clinician workload and reimbursement, alongside the regulatory clearance required for adaptive software. It will also require defining where closed‐loop control complements rather than replaces established interventions such as catheter ablation, which remains a discrete, definitive therapy for many arrhythmias. Future devices should detect performance degradation, route automated decisions to human review, and permit model updates only under supervision, so that long‐term safety and effectiveness are preserved.

## 7 Conclusion

Cardiac arrhythmia management has long been constrained by episodic monitoring and delayed clinical decision‐making, despite major advances in electrophysiology and device‐based therapy. Throughout this review, we have shown that while traditional approaches have improved outcomes, they remain poorly suited to the dynamic and often intermittent nature of arrhythmic disease. The increasing availability of continuous rhythm monitoring and AI‐based signal interpretation has narrowed the detection gap, but meaningful clinical transformation requires moving beyond detection toward integrated systems capable of timely and accountable intervention.

The concept of closed‐loop arrhythmia management represents a natural evolution of existing clinical practice rather than a radical departure from it. Implantable cardiac devices have already demonstrated that automated sensing and therapy can be safe and effective when governed by conservative timing, escalation logic, and clearly defined safety limits. These lessons provide a critical foundation for extending closed‐loop principles to AI‐enabled systems, emphasizing that automation must be structured, predictable, and clinically grounded to improve patient outcomes.

As discussed in this manuscript, the incorporation of artificial intelligence into closed‐loop architectures introduces new opportunities for personalization and adaptability, but also new risks. Adaptive and learning‐based algorithms offer the potential to tailor arrhythmia management to individual patients and evolving physiological states. However, without explicit constraints, supervisory control, and human oversight, such adaptability may compromise stability and safety. The transition from open‐loop monitoring to closed‐loop care therefore depends not on algorithmic sophistication alone, but on careful system design that balances responsiveness with restraint.

Technological feasibility alone is insufficient to ensure clinical success. Closed‐loop AI systems must operate within real‐world environments characterized by noise, uncertainty, and long‐term use. Effective implementation requires robust sensing, reliable inference, controlled actuation, and continuous feedback, all integrated within interoperable clinical workflows. Equally important are mechanisms for transparency, auditability, and ongoing performance evaluation, which allow clinicians to understand, trust, and appropriately supervise automated systems.

Ultimately, closed‐loop AI should be viewed as a tool to augment clinical judgment rather than replace it. When designed and deployed responsibly, such systems have the potential to reduce delays in care, improve personalization, and enhance safety in arrhythmia management. By grounding innovation in established electrophysiologic principles and embedding AI within governed control architectures, closed‐loop systems may represent a sustainable path toward more responsive and patient‐centered arrhythmia care.

## Author Contributions

Y.I.B. conceived and designed the review and takes responsibility for its overall integrity. M.A., B.A., S.B., D.H., M.S., S.S., Y.A., E.S., A.S., and H.S. contributed to the literature search and synthesis within their respective areas of focus. Y.I.B. and M.A. prepared the initial draft, and all remaining authors critically reviewed and revised it for important intellectual content. Y.I.B. prepared the figures and tables and coordinated the revision process. All authors approved the final version of the manuscript and agree to be accountable for all aspects of the work, including its accuracy and integrity.

## Funding

The authors have nothing to report.

## Ethics Statement

Ethical approval is not required for this review article, as it synthesizes previously published studies and does not involve new human or animal subjects.

## Consent

The authors have nothing to report.

## Conflicts of Interest

The authors declare no conflicts of interest.

## Data Availability

Data sharing not applicable to this article as no datasets were generated or analyzed during the current study. This review did not involve the generation or analysis of new data. Consequently, there are no data sets associated with this manuscript.
